# Institutional deliveries and stillbirth and neonatal mortality in the Global Network's Maternal and Newborn Health Registry

**DOI:** 10.1186/s12978-020-01001-x

**Published:** 2020-12-17

**Authors:** Shivaprasad S. Goudar, Norman Goco, Manjunath S. Somannavar, Avinash Kavi, Sunil S. Vernekar, Antoinette Tshefu, Elwyn Chomba, Ana L. Garces, Sarah Saleem, Farnaz Naqvi, Archana Patel, Fabian Esamai, Carl L. Bose, Waldemar A. Carlo, Nancy F. Krebs, Patricia L. Hibberd, Edward A. Liechty, Marion Koso-Thomas, Tracy L. Nolen, Janet Moore, Pooja Iyer, Elizabeth M. McClure, Robert L. Goldenberg, Richard J. Derman

**Affiliations:** 1 KLE Academy of Higher Education and Research Jawaharlal Nehru Medical College, Belagavi, Karnataka India; 2 RTI International, Durham, NC USA; 3 Kinshasa School of Public Health, Kinshasa, Democratic Republic of Congo; 4 University Teaching Hospital, Lusaka, Zambia; 5 Instituto de Nutrición de Centroamérica y Panamá, Guatemala City, Guatemala; 6 Aga Khan University, Karachi, Pakistan; 7 Lata Medical Research Foundation, Nagpur, India; 8 Moi University School of Medicine, Eldoret, Kenya; 9 University of North Carolina at Chapel Hill, Chapel Hill, NC USA; 10 University of Alabama at Birmingham, Birmingham, AL USA; 11 University of Colorado School of Medicine, Denver, CO USA; 12 Boston University School of Public Health, Boston, MA USA; 13 Indiana School of Medicine, University of Indiana, Indianapolis, IN USA; 14 Eunice Kennedy Shriver National Institute of Child Health and Human Development, Bethesda, MD USA; 15 Department of Obstetrics and Gynecology, Columbia University School of Medicine, New York, NY USA; 16 Thomas Jefferson University, Philadelphia, PA USA

**Keywords:** Institutional deliveries, Facility births, Stillbirths, Neonatal mortality, Global network

## Abstract

**Background:**

Few studies have shown how the move toward institutional delivery in low and middle-income countries (LMIC) impacts stillbirth and newborn mortality.

**Objectives:**

The study evaluated trends in institutional delivery in research sites in Belagavi and Nagpur India, Guatemala, Kenya, Pakistan, and Zambia from 2010 to 2018 and compared them to changes in the rates of neonatal mortality and stillbirth.

**Methods:**

We analyzed data from a nine-year interval captured in the Global Network (GN) Maternal Newborn Health Registry (MNHR). Mortality rates were estimated from generalized estimating equations controlling for within-cluster correlation. Cluster-level analyses were performed to assess the association between institutional delivery and mortality rates.

**Results:**

From 2010 to 2018, a total of 413,377 deliveries in 80 clusters across 6 sites in 5 countries were included in these analyses. An increase in the proportion of institutional deliveries occurred in all sites, with a range in 2018 from 57.7 to 99.8%. In 2010, the stillbirth rates ranged from 19.3 per 1000 births in the Kenyan site to 46.2 per 1000 births in the Pakistani site and by 2018, ranged from 9.7 per 1000 births in the Belagavi, India site to 40.8 per 1000 births in the Pakistani site. The 2010 neonatal mortality rates ranged from 19.0 per 1000 live births in the Kenyan site to 51.3 per 1000 live births in the Pakistani site with the 2018 neonatal mortality rates ranging from 9.2 per 1000 live births in the Zambian site to 50.2 per 1000 live births in the Pakistani site. In multivariate modeling, in some but not all sites, the reductions in stillbirth and neonatal death were significantly associated with an increase in the institutional deliveries.

**Conclusions:**

There was an increase in institutional delivery rates in all sites and a reduction in stillbirth and neonatal mortality rates in some of the GN sites over the past decade. The relationship between institutional delivery and a decrease in mortality was significant in some but not all sites. However, the stillbirth and neonatal mortality rates remain at high levels. Understanding the relationship between institutional delivery and stillbirth and neonatal deaths in resource-limited environments will enable development of targeted interventions for reducing the mortality burden.

**Trial registration:**

The study is registered at clinicaltrials.gov. ClinicalTrial.gov Trial Registration: NCT01073475.

## Background

Since 1990, efforts to reduce child mortality have made an impact across the globe. By 2015, the global under-five mortality rate was reduced by 53%, from 91 per 1000 live births to 43 per 1000. However, despite the overall progress in under-five child mortality, less progress was made with neonatal mortality, representing 45% of the 5.9 million under five deaths in 2015 [1]. Furthermore, the burden of death remains unequally distributed, as both sub-Saharan Africa and south Asia recorded a neonatal mortality rate of 29 per 1000 live births, combining for an estimated 2.1 million neonatal deaths recorded in 2015. The burden of stillbirths is similar to that of neonatal mortality and these regions account for a similar proportion of all stillbirths. Most of both neonatal deaths and stillbirths in these regions occur among term or near-term fetuses/neonates. These deaths have been substantially reduced in high-resource settings. To end preventable stillbirths and deaths of newborns and reach the United Nations Sustainable Development Goal (SDG) (3.2) by 2030 (with all countries reducing neonatal mortality to no more than 12 per 1000 live births), success must be achieved in reducing stillbirths and neonatal mortality [2, 3]. Most stillbirths occur during labor and most neonatal deaths occur shortly after delivery [4–7]. Globally, intrapartum-related complications are estimated as the cause of as much as 60% of stillbirths and 23% of neonatal mortality [8]. Skilled birth attendance and an institutional environment capable of providing effective obstetric and neonatal care are needed to significantly reduce stillbirths and neonatal deaths [9, 10]. While delivery in a health facility is assumed to improve birth outcomes, the existing evidence to date has shown contradicting results, particularly in areas where enabling environments are constrained [11–13]. For example, one recent study from Ghana observed that facility delivery was not associated with decreased risk of maternal or neonatal mortality [13].

To date, few prospective studies have assessed the impact of the shift from home births to delivery in health facilities on stillbirths and neonatal mortality across low-resource settings. In a population-based pregnancy registry, we sought to evaluate the trends toward institutional delivery and associated stillbirth and neonatal mortality rates in the *Eunice Kennedy Shriver* National Institute of Child Health and Human Development Global Network for Women’s and Children’s Health Research (GN) sites from January 2010 to December 2018.

## Methods

This study is an analysis of 2010–2018 data from the Maternal Newborn Health Registry (MNHR) of the GN [14]. The MNHR is a prospective, population-based surveillance study of pregnant women and their pregnancy outcomes. From the MNHR, six study sites with complete data over this period were selected for inclusion. These sites included: Eldoret, Kenya; Lusaka, Zambia; Belagavi, Karnataka State and Nagpur, Maharashtra State, India; Thatta, Pakistan; and Chimaltenango, Guatemala. The study population at each site is derived from geographically defined clusters with 300 or more births per year. The MNHR staff enrolled all consenting pregnant women who were residents of the cluster and collected outcome information following delivery and at 42 days postpartum.

Table 1 describes the location, the clusters, and the health institutions contributing to the MNHR for this analysis. The MNHR dataset for this analysis (2010 to 2018) excluded outcomes for women lost to follow-up prior to delivery, medically terminated pregnancies or those resulting in miscarriage, and infants weighing < 1000 g at birth. Early pregnancy losses were excluded because the objective was to assess outcomes associated with facility delivery and those < 1000 g were generally not considered viable in the study facilities. To evaluate trends, we restricted analyses to clusters that contributed to the full study period.

**Table 1:** Site Descriptions

S**ite** (C**oordinating** C**enter**)	L**ocation**	N**o**. **of** C**ontinuing** C**lusters**	N**o**. **and** T**ype of** H**ealth** F**acilities**
**Africa**			
Kenya (Eldoret)	Western region of Kenya in counties of Busia, Bungoma, and Kakamega	16	20 health centers 3 referral hospitals
Zambia (Lusaka)	Kafue and Chongwe districts south and east of Lusaka	10	8 health posts 3 district hospitals 1 tertiary referral hospital
**Latin** **America**			
Guatemala (Chimaltenango)	Western Highlands of Guatemala	11	42 health posts 30 health centers 1 tertiary level referral hospital
**Asia**			
Belagavi, India (Belagavi)	Northwestern corner of state of Karnataka	12	18 primary health centers 8 secondary level hospitals 3 tertiary level hospitals
Nagpur, India (Nagpur)	Located within the state of Maharashtra	19	20 primary health centers 119 sub-centers 10 tertiary level hospitals
Pakistan (Thatta)	Two sub-districts of Thatta district in southern Sindh province	12	47 primary health clinics 25 secondary care facilities 3 tertiary level referral hospitals

Institutional delivery was defined as delivery at either a hospital or clinic (primary health center or community health center). Community deliveries included those that occurred at home (generally the mother’s home or birth attendant’s home). Stillbirth rates were defined as deaths prior to delivery among all births ≥28 weeks gestation or > 1000 g birthweight if gestational age was unavailable (events per 1000 births).

Descriptive analyses were performed. For each mortality outcome, annual estimates, and 95% confidence intervals were obtained from generalized linear models with a Poisson distribution assumption and log link with generalized estimating equations controlling for within-cluster correlation. Parameters in the model include site and year as well as their interaction, with year treated categorically in order not to assume a linear trend over time although a test for a linear trend was completed. Models were also run adjusting for potential confounders including age, maternal education, parity, multiple births, and infant birth weight. Within site estimates were obtained for all deliveries. Cluster-level mortality rates were modeled as a function of institutional delivery rates using repeated measures beta logistic models to account for correlation within clusters across time. The partial correlation coefficient (ρ) between institutional delivery rates and each mortality outcome was also calculated accounting for the repeated measures within cluster across time in order to assess the association between the institutional delivery rate and the outcome, while accounting for the potential confounders. All analyses were conducted using SAS v.9.4 (Cary, NC).

## Results

**Fig. 1:**
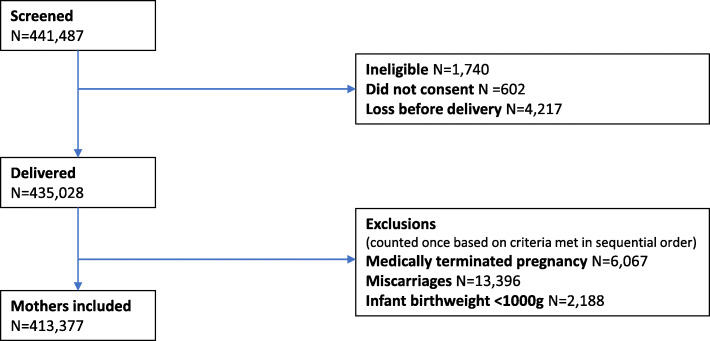
CONSORT diagram - Enrollment and deliveries

From 2010 to 2018, a total of 413,377 deliveries were included in these analyses (Fig. 1), with a range across sites of 58,686 deliveries in the Guatemalan site to 79,011 deliveries in the Nagpur site (Table 2). Overall, 82.6% of the pregnant women in this cohort were between the ages of 20–35, with the Kenyan (22.2%) and Zambian (24.6%) sites having the largest proportion of women below the age of 20. Approximately 80% of women had a primary level of education or higher. However, the Pakistani site reported that 85.8% of women had no formal education. Regarding parity, nearly 30% of the cohort in the Kenyan, Zambian and Guatemalan sites, greater than 40% in the Belagavi and Nagpur sites, and less than 20% in the Pakistani site were nulliparous. In contrast, more than 30% of the women in the Kenyan, Zambian, and Guatemalan sites and nearly half in the Pakistani site reported two or more prior births, while the Belagavi and Nagpur sites reported that less than 10% of the women had two or more prior births. The proportion of women with at least one antenatal care visit was nearly 100% for all sites except for the Pakistani site, which was just below 90%. Among all sites, between 47 and 50% of infants were female and less than 3% were the result of a multiple gestation. The African sites reported the greatest proportion of infants with a birthweight ≥2500 g (96.1% for the Kenyan site and 93.5% for the Zambian site), while the other sites reported > 2500 g birth weight rates between 78.9 and 84.0%.

**Table 2:** Maternal and infant demographic and clinical characteristics by site

	Kenya	Zambia	Guatemala	Belagavi	Nagpur	Pakistan
Deliveries	73,896	62,382	58,686	78,916	79,011	60,486
Maternal Age, N (%)						
< 20	16,274 (22.2)	15,310 (24.6)	9792 (16.7)	8437 (10.7)	1718 (2.2)	2187 (3.6)
20–35	53,873 (73.5)	42,002 (67.4)	42,775 (72.9)	70,270 (89.1)	76,945 (97.4)	54,746 (90.7)
> 35	3127 (4.3)	5015 (8.0)	6109 (10.4)	186 (0.2)	324 (0.4)	3455 (5.7)
Maternal Education, N (%)						
No formal education	1653 (2.3)	5445 (8.8)	10,051 (17.1)	12,629 (16.1)	2491 (3.2)	51,831 (85.8)
Primary or Secondary	67,043 (91.5)	55,746 (89.6)	46,156 (78.7)	58,340 (74.3)	62,608 (79.3)	7588 (12.6)
University +	4598 (6.3)	1007 (1.6)	2472 (4.2)	7579 (9.6)	13,828 (17.5)	968 (1.6)
Parity, N (%)						
0	21,236 (29.0)	18,103 (29.0)	16,863 (28.7)	32,053 (40.8)	38,783 (49.1)	11,028 (18.7)
1–2	28,495 (38.9)	23,968 (38.4)	22,329 (38.1)	41,439 (52.7)	38,384 (49.6)	19,544 (33.1)
> 2	23,570 (32.2)	20,279 (32.5)	19,491 (33.2)	5068 (6.5)	1810 (2.3)	28,403 (48.2)
At least one antenatal care visit	72,430 (98.0)	62,184 (99.7)	56,422 (96.2)	78,884 (100)	78,841 (99.8)	54,013 (89.4)
Births	74,814	62,999	59,051	79,479	79,628	61,187
Infant Gender, N (%)						
Male	37,730 (50.5)	32,416 (51.5)	29,993 (50.9)	41,011 (51.6)	41,374 (52.2)	31,591 (52.0)
Female	36,987 (49.5)	30,518 (48.5)	28,987 (49.1)	38,403 (48.4)	37,953 (47.8)	29,183 (48.0)
Infant Birthweight, N (%)						
1000–1499 g	333 (0.4)	386 (0.6)	406 (0.7)	874 (1.1)	1027 (1.3)	1208 (2.0)
1500–2499 g	2594 (3.5)	3684 (5.9)	9016 (15.3)	12,779 (16.1)	12,857 (16.2)	11,593 (19.1)
≥ 2500 g	71,743 (96.1)	58,863 (93.5)	49,543 (84.0)	65,779 (82.8)	65,377 (82.5)	47,950 (78.9)
Multiple Birth, N (%)						
Yes	1790 (2.4)	1214 (1.9)	720 (1.2)	1121 (1.4)	1226 (1.5)	1372 (2.3)
No	72,970 (97.6)	61,754 (98.1)	58,274 (98.8)	78,325 (98.6)	78,266 (98.5)	59,425 (97.7)

MNH Registry 2010–2018 deliveries excluding women lost to follow-up prior to delivery, miscarriages/medical terminations and births < 1000 g. Infant birthweight includes measured and estimated values

There were substantial increases in institutional deliveries observed for all sites (Table 3), with the largest increases at the Kenyan site (35.8 to 86.7% for 2010 to 2018), the Zambian site (50.5 to 88.2%), the Pakistani site (46.1 to 72.5%), and the Guatemalan site (28.2 to 57.7%). The proportion of institutional deliveries in the two Indian sites were already above 90% in 2010 and increased to nearly 99% or greater by 2018 (Belagavi: 92.7 to 98.7%; Nagpur: 90.0 to 99.8%). Trends in the proportion of deliveries attended by a trained health worker also increased over time and were generally consistent with the rates for institutional deliveries (data not shown). While rates of caesarean deliveries also increased over time, they were generally low for the African sites (< 3% across all years), rose from 6 to 15% in the Pakistani site, and were greater than 10% in 2010 increasing to between 28 and 38% in 2018 for the Indian and Guatemalan sites, respectively (data not shown).

**Table 3:** Trends in Delivery and Mortality Rates by Site

Year	Overall Births, N	Institutional Delivery N (%)	Overall N (95% CI)	
			Stillbirth rate (per 1000 births)	Neonatal mortality < 28 days rate (per 1000 live births)
**Kenya**				
2010	9177	3242 (35.8)	19.3 (14.6, 25.5)	19.0 (13.3, 27.0)
2011	9523	3672 (39.0)	16.6 (12.2, 22.5)	12.3 (9.8, 15.3)
2012	8905	3690 (41.9)	25.6 (20.1, 32.6)	13.6 (10.8, 17.2)
2013	8460	4236 (50.8)	19.5 (14.9, 25.6)	13.8 (11.0, 17.5)
2014	7875	5037 (64.8)	23.0 (18.3, 28.7)	11.5 (8.9, 14.9)
2015	7761	5560 (72.5)	21.7 (17.1, 27.5)	11.0 (8.4, 14.5)
2016	7692	5890 (77.5)	20.1 (16.6, 24.3)	12.6 (9.8, 16.3)
2017	7729	5386 (70.6)	22.6 (19.3, 26.5)	13.6 (9.9, 18.8)
2018	7692	6587 (86.7)	15.8 (12.6, 19.7)	11.8 (8.4, 16.5)
Birth-Level Analyses: Adjusted Linear trend *p*-value^1^			0.4926	0.1419
Cluster-Level Analyses of mortality outcome with institutional delivery rate (IDR)				
Odds ratio for 1 unit increase in IDR (*p*-value)			0.87 (0.5165)	
Partial correlation coefficient with IDR (*p*-value)			−0.04 (0.6299)	−0.18 (0.0414)
**Zambia**				
2010	7137	3571 (50.5)	24.2 (18.2, 32.1)	22.8 (17.5, 29.6)
2011	7113	4070 (57.7)	17.5 (12.5, 24.6)	15.9 (12.4, 20.4)
2012	6780	4458 (66.3)	18.9 (14.6, 24.6)	16.6 (12.3, 22.5)
2013	6798	4666 (69.3)	18.7 (14.4, 24.4)	15.2 (11.8, 19.7)
2014	6671	5171 (78.2)	13.6 (10.3, 17.9)	13.4 (8.8, 20.5)
2015	7265	5936 (82.7)	13.4 (8.7, 20.7)	13.9 (10.5, 18.4)
2016	7322	6054 (83.5)	16.6 (13.3, 20.7)	16.1 (12.8, 20.2)
2017	7074	6048 (86.6)	16.8 (13.4, 21.2)	13.2 (9.4, 18.5)
2018	6839	5968 (88.2)	16.0 (12.5, 20.5)	9.2 (6.9, 12.3)
Birth-Level Analyses: Adjusted Linear trend *p*-value^1^			0.9896	0.0005
Cluster-Level Analyses of mortality outcome with institutional delivery rate (IDR)				
Odds ratio for 1 unit increase in IDR (*p*-value)			0.59 (0.0704)	0.45 (0.0092)
Partial correlation coefficient with IDR (*p*-value)			−0.23 (0.0398)	−0.28 (0.0127)
**Guatemala**				
2010	4042	1134 (28.2)	20.7 (16.5, 26.0)	27.5 (21.4, 35.4)
2011	5900	2023 (34.5)	19.5 (15.4, 24.7)	22.4 (18.2, 27.5)
2012	5843	2422 (41.7)	15.2 (11.2, 20.7)	17.3 (13.6, 22.1)
2013	6761	3234 (48.1)	17.3 (13.8, 21.8)	22.0 (18.6, 25.9)
2014	7010	3447 (49.5)	14.5 (11.7, 18.0)	29.4 (25.8, 33.5)
2015	7326	3710 (51.0)	18.6 (14.0, 24.9)	25.2 (21.1, 30.0)
2016	7905	3941 (50.2)	19.9 (15.4, 25.6)	26.9 (23.3, 31.0)
2017	7410	4013 (54.4)	18.9 (14.7, 24.3)	20.0 (15.7, 25.4)
2018	6854	3931 (57.7)	16.5 (13.8, 19.7)	21.9 (18.3, 26.3)
Birth-Level Analyses: Adjusted Linear trend *p*-value^1^			0.4570	0.0324
Cluster-Level Analyses of mortality outcome with institutional delivery rate (IDR)				
Odds ratio for 1 unit increase in IDR (*p*-value)				0.68 (0.0859)
Partial correlation coefficient with IDR (*p*-value)			−0.40 (0.0002)	−0.12 (0.2816)
**Belagavi, India**				
2010	11,922	10,961 (92.7)	21.5 (17.7, 26.0)	24.4 (20.3, 29.2)
2011	12,263	11,559 (94.9)	20.7 (16.5, 26.0)	21.9 (16.3, 29.4)
2012	12,831	12,173 (95.7)	20.1 (17.3, 23.3)	21.3 (18.0, 25.1)
2013	11,793	11,245 (96.1)	15.0 (12.8, 17.6)	23.6 (20.7, 26.9)
2014	6942	6529 (94.8)	15.8 (12.7, 19.6)	22.0 (19.1, 25.4)
2015	6637	6360 (96.6)	14.1 (10.8, 18.3)	18.5 (16.1, 21.3)
2016	6254	6086 (98.0)	13.6 (10.7, 17.2)	16.6 (13.9, 19.7)
2017	5641	5492 (97.9)	11.2 (9.6, 13.0)	18.1 (16.4, 20.1)
2018	5196	5079 (98.7)	9.7 (7.3, 13.0)	13.0 (10.1, 16.7)
Birth-Level Analyses: Adjusted Linear trend *p*-value^1^			0.0049	0.0243
Cluster-Level Analyses of mortality outcome with institutional delivery rate (IDR)				
Odds ratio for 1 unit increase in IDR (*p*-value)			0.04 (0.0006)	0.08 (0.0018)
Partial correlation coefficient with IDR (*p*-value)			−0.33 (0.0011)	−0.27 (0.0083)
**Nagpur, India**				
2010	9719	8671 (90.0)	28.6 (24.9, 33.0)	23.5 (19.8, 27.8)
2011	9203	8823 (96.6)	20.7 (18.0, 23.9)	17.9 (14.7, 21.9)
2012	9184	8939 (98.1)	22.0 (19.0, 25.6)	21.7 (18.2, 25.9)
2013	9568	9364 (98.8)	20.7 (18.0, 23.9)	23.5 (19.8, 27.8)
2014	8762	8628 (99.1)	19.6 (16.0, 24.0)	18.7 (15.6, 22.4)
2015	9469	9335 (99.5)	19.4 (16.2, 23.2)	17.7 (15.3, 20.6)
2016	8699	8557 (99.5)	17.8 (14.4, 21.9)	20.6 (18.2, 23.2)
2017	7987	7913 (99.8)	16.5 (13.6, 19.9)	16.5 (14.1, 19.3)
2018	7037	6971 (99.8)	11.5 (8.8, 15.1)	18.0 (15.4, 21.2)
Birth-Level Analyses: Adjusted Linear trend *p*-value^1, 2^			<.0001	
Cluster-Level Analyses of mortality outcome with institutional delivery rate (IDR)				
Odds ratio for 1 unit increase in IDR (*p*-value)			0.04 (<.0001)	0.22 (0.0302)
Partial correlation coefficient with IDR (*p*-value)			−0.42 (<.0001)	−0.17 (0.0369)
**Pakistan**				
2010	8088	3677 (46.1)	46.2 (39.3, 54.1)	51.3 (42.7, 61.5)
2011	8120	4002 (50.0)	51.4 (43.0, 61.4)	58.3 (50.8, 66.8)
2012	6890	3455 (50.8)	49.1 (43.6, 55.4)	46.5 (40.0, 54.1)
2013	7033	4002 (57.7)	59.5 (50.1, 70.8)	51.7 (45.8, 58.4)
2014	6844	4105 (60.6)	50.9 (44.6, 58.2)	45.0 (41.0, 49.4)
2015	6396	3955 (62.7)	47.0 (40.4, 54.7)	52.8 (45.9, 60.7)
2016	6089	3910 (65.0)	43.1 (36.6, 50.8)	48.3 (43.4, 53.7)
2017	5958	4014 (68.1)	42.1 (37.5, 47.3)	48.3 (43.1, 54.1)
2018	5769	4137 (72.5)	40.8 (35.5, 46.9)	50.2 (45.2, 55.7)
Birth-Level Analyses: Adjusted Linear trend *p*-value^1^			<.0001	0.0011
Cluster-Level Analyses of mortality outcome with institutional delivery rate (IDR)				
Odds ratio for 1 unit increase in IDR (*p*-value)			0.50 (0.0015)	0.86 (0.4499)
Partial correlation coefficient with IDR (*p*-value)			−0.23 (0.0259)	−0.07 (0.4756)

^1^ Adjusted linear models control for the following characteristics: maternal age, maternal education, parity, multiple birth, and categorized infant birth weight

^2^ The adjusted model for GN11 Nagpur failed to converge due to a low number of cases among Community birth

**Fig. 2:**
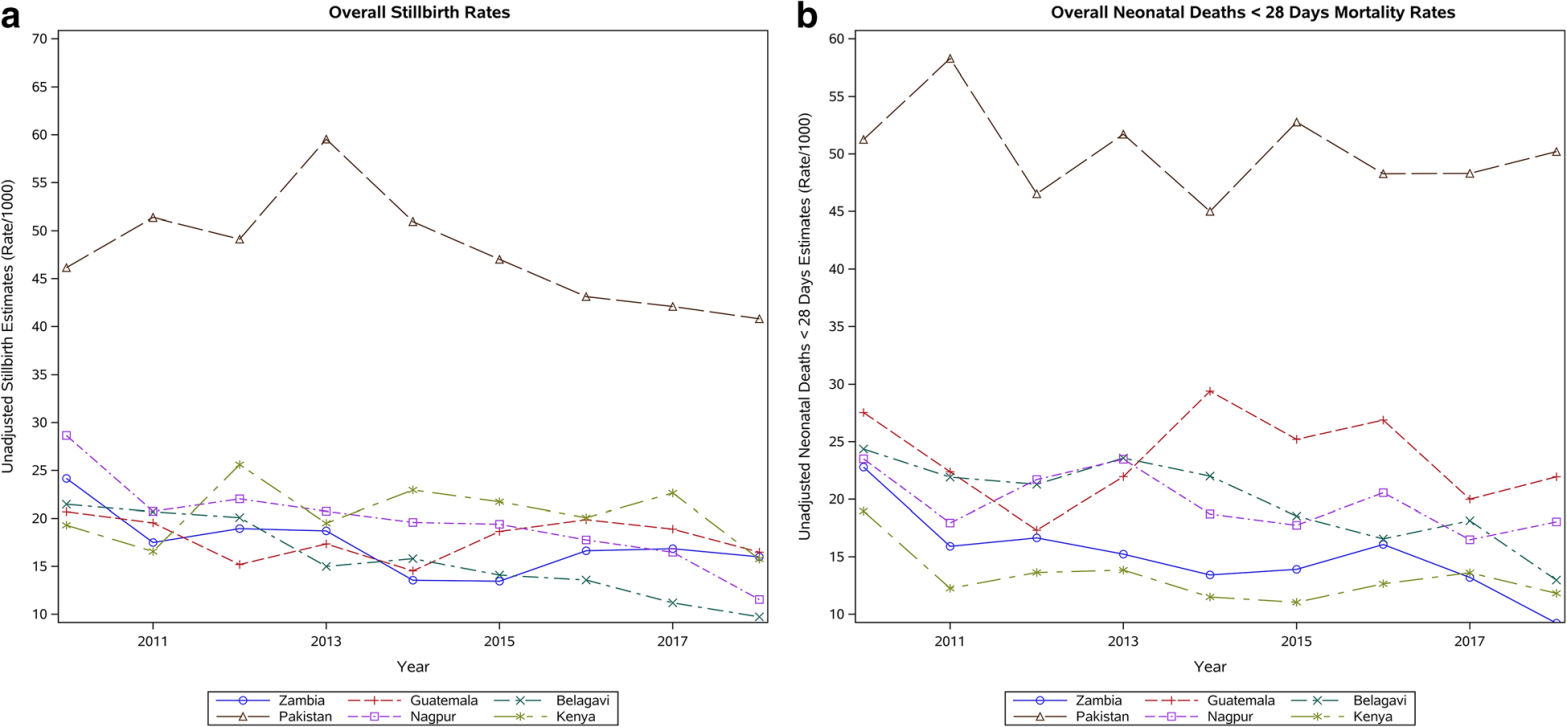
Stillbirth and neonatal mortality rates - trends over time by country site

Figure 2 displays stillbirth and neonatal mortality rates for each site by year. In general, the overall rates for each outcome decreased over time for most sites, with year-to-year fluctuation. The Pakistani site consistently reported the highest stillbirth and neonatal mortality rates.

Table 3 presents results for trends in stillbirth and neonatal mortality rates over time. In the unadjusted analyses, every site showed at least a modest reduction of stillbirth rates from 2010 to 2018. When adjusting for covariates, the test for linear trend over time was significant for stillbirths only for the Asian sites, but not for the African or Guatemalan sites. Comparing 2010 to 2018, every site had at least a small reduction in the neonatal mortality rates. In the adjusted analyses, neonatal mortality was reduced significantly for the Zambian, Pakistani, Guatemalan and Belagavi sites. In the Nagpur site, the overall unadjusted stillbirth rate fell from 28.6 to 11.5 per 1000 (*p* < 0.0001); however, the adjusted model for neonatal mortality failed to converge.

In the cluster-level analyses to assess the association between institutional delivery rates and stillbirth rates (Table 3), evidence for an association was observed for stillbirths for the Zambian, Belagavi, Nagpur, Guatemalan and Pakistani sites. Neonatal mortality rates were associated with increases in institutional delivery for the Kenyan, Zambian, Belagavi, and Nagpur sites. In each of these sites, the partial correlation coefficients (ρ) between institutional delivery rates and stillbirths and/or neonatal mortality rates were negative and the odds ratios (OR) for a 1 unit increase in institutional delivery were less than one, suggesting that stillbirth and/or neonatal mortality rates were lower when institutional delivery rates were higher. However, generally these results suggest that the observed associations between institutional delivery and mortality outcomes were relatively small.

## Discussion

This study analyzed 9 years (2010 to 2018) of pregnancy and delivery outcome data from six sites within the GN MNHR. The proportion of institutional deliveries increased across all GN sites. The shift was associated with some statistically significant decreases in stillbirth and neonatal mortality over the same period; however, overall, these changes were modest. Among the African sites, a large increase in institutional births was observed in the Zambian site and at the cluster level, higher institutional birth rates were associated with lower stillbirth and neonatal mortality rates. However, in Kenya, a large increase in institutional deliveries was also observed, with no significant association between institutional delivery rates and stillbirths and neonatal mortality rates.

The proportion of institutional deliveries in the Guatemalan site doubled over time, yet in 2018 institutional deliveries still accounted for less than 60% of all deliveries. There was, however, no significant decrease in stillbirth rates over time. Along with the increase in institutional deliveries, a significant decrease in the stillbirth and neonatal mortality rates was observed, but with year-to-year fluctuation.

Among the Asian sites, smaller increases in institutional deliveries were observed in the two Indian sites - Belagavi and Nagpur - with greater than 90% of births occurring in health facilities in 2010 and greater than 98% by the end of 2018. For both Indian sites, lower stillbirth and neonatal mortality rates were associated with higher institutional delivery rates. In the Pakistani site, the proportion of institutional deliveries moderately increased from 2010 to 2018 and there were significant decreases in the stillbirth and neonatal mortality rates. However, there was only a significant association between stillbirth rates and institutional delivery rates.

Prior and during the time of this study, the governments of many low and middle-income countries (LMIC) have encouraged institutional delivery through incentive programs (e.g., conditional cash transfers and removal of user fees) as a strategy to improve maternal and newborn outcomes [15, 16]. Evaluations of these strategies have demonstrated varied results in increasing institutional deliveries [17–19].

A recently published study of the major determinants of facility birth on mortality outcomes through a secondary analysis of surveillance data in Ghana found that facility delivery did not result in improved survival for newborns [13]. Investigators assessed the quality of care of obstetric and newborn services among the facilities in the study area. The study highlighted the need to improve the quality of obstetric and newborn care in order to reduce mortality. Some studies have shown that institutional deliveries may reduce perinatal mortality attributed to intrapartum complications [20] and may reduce neonatal mortality [17]. In our study, we observed an increase in institutional deliveries at each of the research sites and identified an associated decrease in neonatal mortality in four of the six sites. Despite these improvements, the neonatal mortality rates remain at high levels when compared with corresponding rates in high-income counties [21]. Further progress may require an enhanced focus on improving the quality of delivery services as well as a corresponding improvement in the overall health systems in LMICs.

Our study includes a significant number of observations and 9 years of data enabling us to measure trends over time. The MNHR incorporates rigorous training of data collectors and close, routine monitoring to help ensure high-quality data. The database for this study included over 400,000 pregnancies and corresponding delivery outcomes to provide a strong framework to analyze trends in institutional delivery and mortality. However, our analyses were limited by the number of factors included in the study and the consistency of variables collected over the study period. This reduced our capacity to explore in depth the associations between institutional deliveries and outcomes.

## Conclusions

While we did observe important increases in institution-based deliveries, these increases were not consistently associated with decreased stillbirth or neonatal mortality rates. Both stillbirth and neonatal mortality rates remain high in these settings, despite the increases in facility delivery. Research on the factors associated with stillbirth and neonatal mortality in institutional deliveries is needed to develop interventions aimed at reducing those mortalities.

## Data Availability

Data from the study will be available at the NICHD data repository (N-DASH): https://dash.nichd.nih.gov/.

## Abbreviations

GN: Global Network for Women’s and Children’s Health Research
JSY: Janani Suraksha Yojana
LMIC: Low and middle-income country
MNHR: Maternal and newborn health registry
MDG: Millennium Development Goals
NMR: Neonatal mortality rate
SDG: Sustainable Development Goal
